# Generation of a helper phage for the fluorescent detection of peptide-target interactions by dual-display phages

**DOI:** 10.1038/s41598-023-45087-2

**Published:** 2023-11-02

**Authors:** Laura Maria De Plano, Salvatore Oddo, Salvatore P. P. Guglielmino, Antonella Caccamo, Sabrina Conoci

**Affiliations:** 1 Department of Chemical, Biological, Pharmaceutical and Environmental Sciences, University of Messina, Viale F. Stagno d’Alcontres 31, Messina, Italy; 2 Department of Chemistry G. Ciamician, University of Bologna, Via F. Selmi 2, Bologna, Italy; 3 LAB Sense Beyond Nano–DSFTM CNR, Viale F. Stagno d’Alcontres 31, Messina, Italy; 4 CNR Institute for Microelectronics and Microsystems, Strada VIII, 5, Catania, Italy; 5 STMicroelectronics, Stradale Primosole 50, 95121 Catania, Italy

**Keywords:** Molecular biology, Biotechnology

## Abstract

Phage display is a molecular biology technique that allows the presentation of foreign peptides on the surface of bacteriophages. It is widely utilized for applications such as the discovery of biomarkers, the development of therapeutic antibodies, and the investigation of protein–protein interactions. When employing phages in diagnostic and therapeutic monitoring assays, it is essential to couple them with a detection system capable of revealing and quantifying the interaction between the peptide displayed on the phage capsid and the target of interest. This process is often technically challenging and costly. Here, we generated a fluorescent helper phage vector displaying sfGFP in-frame to the pIII of the capsid proteins. Further, we developed an exchangeable dual-display phage system by combining our newly developed fluorescent helper phage vector with a phagemid vector harboring the engineered pVIII with a peptide-probe. By doing so, the sfGFP and a peptide-probe are displayed on the same phage particle. Notably, our dual-display approach is highly flexible as it allows for easy exchange of the displayed peptide-probe on the pVIII to gain the desired selectivity, while maintaining the sfGFP gene, which allows easy visualization and quantification of the interaction peptide-probe. We anticipate that this system will reduce time and costs compared to the current phage-based detection systems.

## Introduction

Phage display is a molecular technique that utilizes bacteriophages to select peptides or proteins of interest^1^. One commonly used bacteriophage in phage display is the M13 phage, which has proven to be highly effective for this purpose^2^. By genetically manipulating the M13 phage to display a specific peptide or protein on its surface, phage display enables the selection of phage variants that bind to a target molecule with high affinity and specificity^3^. This technique has been widely utilized for various applications, including antibody discovery, protein–protein interaction studies, epitope mapping, drug development, and identification of biomarkers^4–7^.

The M13 phage consists of a long filamentous structure with a single-stranded, 6400 bp DNA genome enclosed within a protein coat. The phage particle consists of a long cylindrical protein structure, 1 µm in length and 7 nm in diameter^8^. The coat of the M13 phage is composed of several different coat proteins that play essential roles in the phage's life cycle and its ability to display foreign peptides or proteins. The primary coat protein of the M13 phage is called pVIII, and it forms the majority of the coat, accounting for approximately 2700 copies per phage particle^9^. The pVIII protein is responsible for the elongated shape of the phage and provides structural stability to the virion^10^. The pIII protein, also known as the adsorption protein, plays a key role in the infection process of the M13 phage^11^. It facilitates the attachment of the phage to the bacterial host by binding to specific receptors on the bacterial surface. This interaction is essential for the successful infection of the host bacterium and subsequent replication of the phage.

In phage display, the pIII and pVIII proteins are often exploited for presenting foreign peptides or proteins^12^. By genetically engineering the M13 phage, the genes encoding these two proteins can be modified by introducing a sequence of DNA coding the peptide of interest, thereby fusing the foreign peptide or protein to the N-terminus of the pIII or pVIII proteins. This allows the displayed molecule to be presented on the surface of the phage particle.

Two classes of vectors are used for phage display: the phagemid vector and the phage vector^13,14^. The former allows for the expression of large fusions with any phage capsid proteins. However, it exhibits a low efficiency of incorporation into the produced phage particle. The latter involves direct modification of the M13 phage genome. In this case, every copy of the recombinant capsid protein results in recombination in the phage progeny. Nonetheless, it should be noted that the length of the added foreign molecule is closely related to the capsid protein being modified. To this end, pVIII phage display libraries are mostly limited to sizes of up to 10 amino acids as longer insertions rarely assemble in phage particles^15^. Large sequences have mainly been fused to minor capsid proteins using both phagemid and phage vectors^16^.

During the last few years, the utilization of engineered phages has significantly surged given their tolerance for a wide range of temperatures, solvents and radiation that is not available in other biological molecules (e.g., antibodies). This makes engineered phages excellent candidates for several diagnostic and therapeutic systems^17^. However, their use needs to be associated with detection systems that permit to visualize the interaction between a phage and its target^18^. Currently, researchers routinely use techniques such as ELISAs, PCR for gene detection, and chemical-physical signals for these purposes^19–21^. Moreover, functional groups naturally expressed on phage capsids are used for electrostatic adsorption or bioconjugation with a responsive surface or fluorophore to give an optical signal^22–24^. Although sensitive, these assays are laborious and expensive and can give altered positive results. In this work, we developed a fluorescent helper phage vector and used it to build a dual display phage system, which displays sfGFP fused to the pIII, and a peptide-probe fused to the pVIII. While we performed proof-of-concept studies in *P. aeruginosa* and the SH-SY5Y cell line, the system is highly flexible and by simply exchanging the peptide-probe can be used to identify any other target.

## Results

### Cloning of sfGFP into pIII

**Figure 1:**
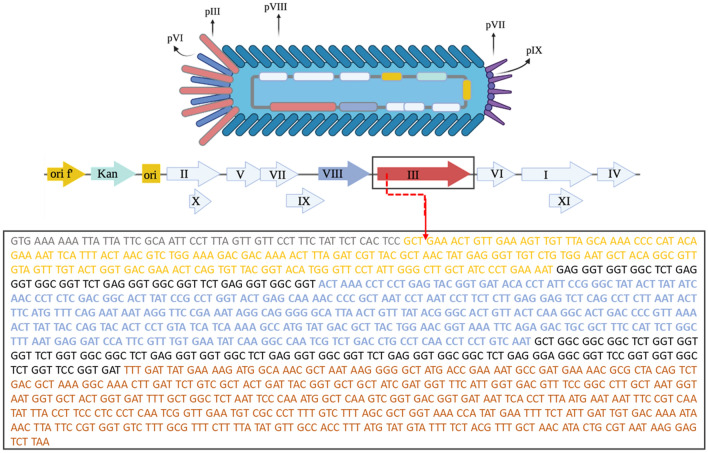
Structural organization of the M13K07 phage. Schematic representation of the structure of the M13K07 phage (top) with its linear ssDNA highlighting its genes (middle). Nucleotide sequence of gene-3 (bottom). The gene-3 sequence codes for a peptide signal (grey), N1 (yellow) and N2 (blue) domains involved in the bacteria infection, and the C terminal domain (orange) required for the release of viral particles from the host bacterial membrane. The two sequences in black represent two glycine-rich linker regions. The red arrow indicates the point of insertion of the sfGFP gene sequence.

To create a fluorescence phage vector, we used the M13K07 phage as a starting point. This specific phage originates from the M13 wild-type phage, and it harbors a p15A origin of replication of *E. coli* and a kanamycin resistance gene (Fig. 1). These genomic additions allow it to replicate inside a bacteria host and grow in the presence of kanamycin, respectively. Notably, despite this extra genetic material, the phenotype of the M13K07 phage is the same as that of the M13 wild type phage. Specifically, the pVIII protein is displayed on the capsid’s entire length, while the other minor proteins (pIII, pVII, pIX, pVI) are present in the terminal ends of the capsid structure (Fig. 1).

To achieve our objective, we cloned the gene coding for the super folder green fluorescence protein (sfGFP) within gene3*,* which encodes the pIII protein. Notably, when modifying gene3, it is crucial to insert the exogenous sequence in frame with its coding region without interfering with the function of the pIII protein. *Gene3* of the M13K07 phage codes for an immature pIII, which is 424 amino acids long^25^. The first 18 amino acids of the immature pIII represent a signal peptide, which eventually is removed to form a mature pIII protein of 406 amino acids^25^. The mature pIII protein is divided into two N-terminal domains (N1 and N2), which are involved in the penetration and adsorption of the phage during infection (reported in black Fig. 1). N1 and N2 are linked by a glycine-rich region. A second glycine-rich region divides N2 from the C-terminal domain of the protein, which is responsible for the interaction with the pVI allowing the anchoring of the phage capsid^11^. Overall, the correct folding of pIII is fundamental for the assembly and release of the filamentous phage from the bacteria host^26,27^. To avoid interfering with the arrangement and the functionality of the pIII, we decide to add the cDNA coding for sfGFP downstream of the codon that codified for the first amino acids of the mature protein (Fig. 1). By doing so, all the recombinant pIII proteins generated by engineered *gene3* carry the sfGFP protein without altering the function of the protein itself.

**Figure 2:**
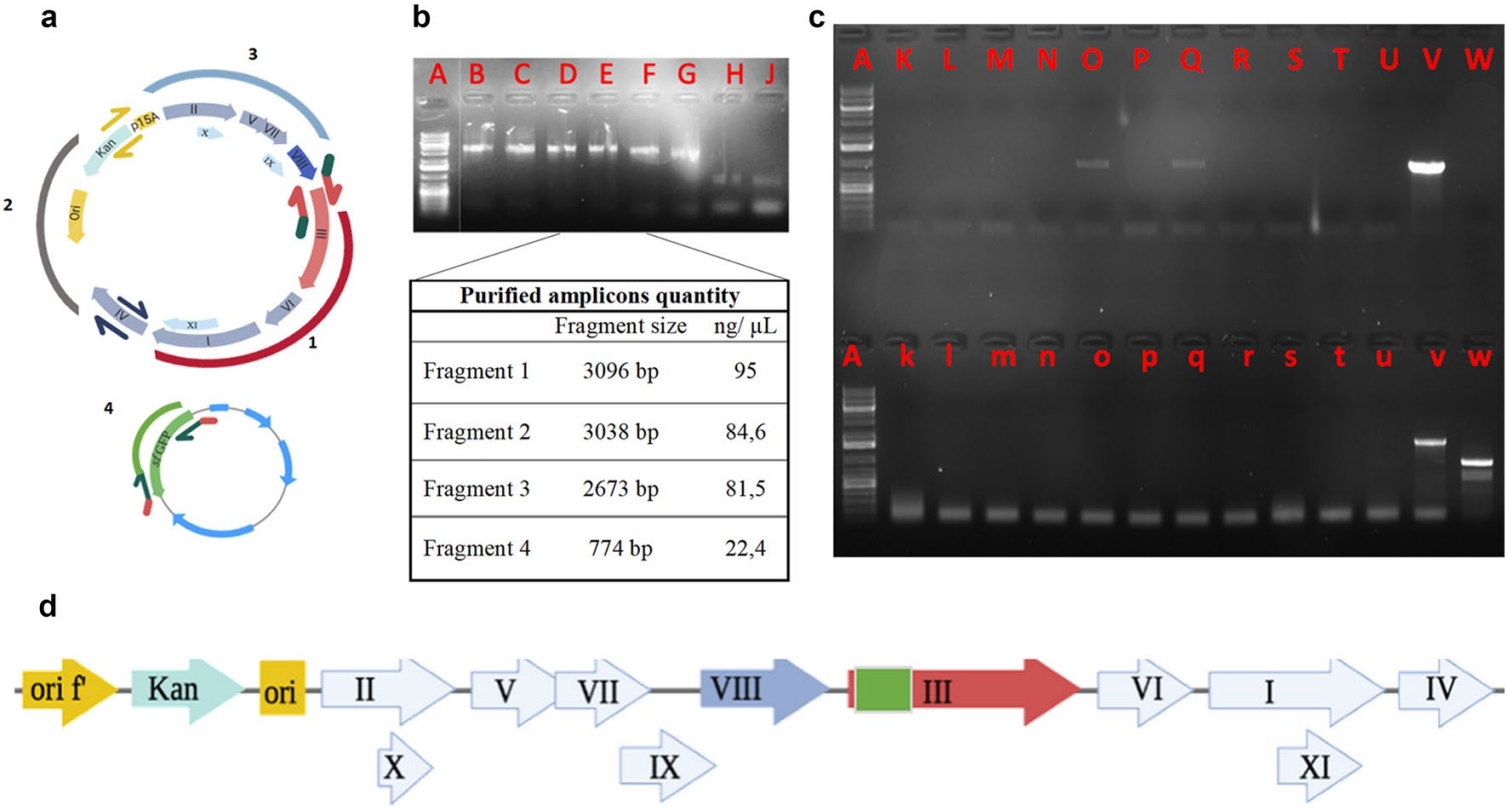
Generation of a pIII-sfGFP fusion protein. (**a**) Cartoon representation of the genome structure of the two systems used for the cloning; the arrows indicate the location and orientation of the primers used. The predictive fragments that will derive from the PCR are depicted in red (fragment 1), grey (fragment 2), light blue (fragment 3), and green (fragment 4). (**b**) Electrophoresis gel of the PCR products shows the obtained amplicon bands (red letters: A DNA marker, B and C fragment 1, D and E fragment 2, F and G fragment 3, H and J fragment 4). After purification, the amplicons were quantified, and their concentration is reported next to the band sizes. (**c**) Electrophoresis gel picture of 13 PCR colonies obtained after the cloning and performed to check the integration of sfGFP (red letter: from K to W) and the correct circularization of the new genome construct (red letter: from k to w; A indicates the DNA marker). The DNA from the amplicon in lanes v and V was isolated and sequenced. (**d**) Representation of the final modified phage genome.

To clone the sfGFP gene within *gene3*, we design four primer pairs, each with specific 5′ overhangs (~ 24–27 bp long; Table 1). The length of the overhangs is sufficient to anneal efficiently during the ligation reaction^28^. Three primer pairs were used to amplify the M13K07 genome; by doing so, we obtained three different fragments named 1–3 in Fig. 2a,b, each of about 3 Kb. The remaining primer pair was used to amplify the sfGFP gene, referred to as fragment four in Fig. 2a,b. The sequence of each overhang is complementary to the overhang of the primer used to amplify the adjacent fragment of the viral genome. For example, fragment 2 of the viral genome was amplified using a forward primer with a 5′ overhang complementary to the 5′ overhang of the reverse primer used to amplify fragment 1 (Fig. 2a). The overhangs of the reverse primer used to amplify sfGFP (fragment four) were complementary to the overhang of the forward primer used to amplify fragment 3. In contrast, the forward primer for the sfGFP was complementary to the reverse primer used for fragment one (Fig. 2a,b).

**Table 1:** Primers used in the cloning process.

Primers design			
Name		Sequence 5′→3′	Location
Fragment 1	LDP018 FW-1	gaggatttagaagtattagactttacaaacaattcgacaac	M13K07 genome
	LDP072 RV-1	cacggcatggatgaactctacaaagaaactgttgaaagttgtttagcaaaacccca	
Fragment 2	LDP019 FW-2	agttgtcgaattgtttgtaaagtctaatacttctaaatcctc	
	LDP020 RV-2	tttatccgtactcctgatgatgcatggttactcacc	
Fragment 3	LDP021 FW-3	tggtgagtaaccatgcatcatcaggagtacggataaa	
	LDP069 RV-3	ggtgaacagttcttcacctttactcatagcggagtgagaatagaaaggaaca	
Fragment 4	LDP071 FW-4	gggttttgctaaacaactttcaacagtttctttgtagagttcatccatgccgtg	Plasmid-*sf*GFP
	LDP070 RV-4	ttagttgttcctttctattctcactccgctatgagtaaaggtgaagaactgttcacc	

The primers were reported in the orientation 5’-3’ and the starting genome was also reported.

After we amplified the four fragments with a high-fidelity polymerase, we used the Gibson assembly mix^28^ to ligate together all four fragments (Fig. 2b). We then transformed *E. coli* with the newly generated phage vector containing the sfGFP and selected the proper clones by kanamycin resistance. The kanamycin-resistant clones were amplified with the sfGFP primers to check the presence of such gene. Of the selected clones, only three presented a band corresponding to the sfGFP gene, and among these, one confirmed the correct circularization of phage genome (Fig. 2c). This was confirmed by further amplification of the regions overlapping the various segments. We then validated the identity of the helper phage containing the sfGFP gene (herein referred to as M13K-GFP) by sequencing the whole genome. The final structure of the newly generated helper phage is shown in Fig. 2d).

### Characterization of the new M13K-GFP help phage

**Figure 3:**
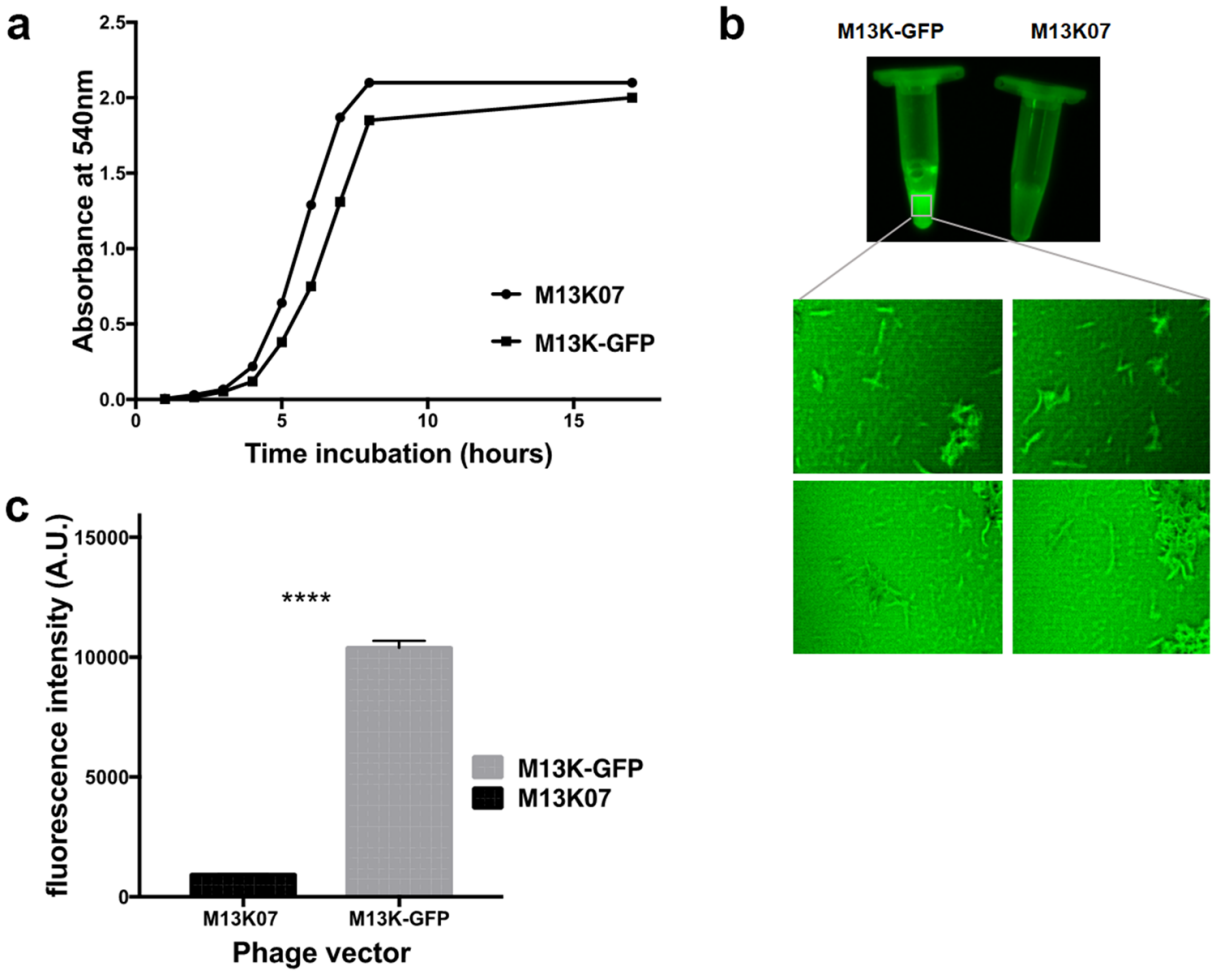
Characterization of M13K-GFP phage vector. (**a**) The picture shows the growth of *E. coli* infected with M13K07 or M13K-GFP, which was monitored every hour for 8 h, then again after 16 h by absorbance at 540 nm. (**b**) Picture of the tube with M13K-GFP and M13K07 phage solutions captured by Azura Fluorescent Imaging System under RGD light and the confocal-fluorescent microscopy photos of M13K-GFP using a FITC filter. (**c**) Fluorescence emission of 1 × 10^10^ TU/mL M13K-GFP and M13K07 phage by multiscan Vector 3 system. Paired t-test indicates a significant difference (p < 0.0001).

We then compared the infective capabilities of M13K-GFP with those of the wildtype M13K07 phage vector. To do so, we first infected *E. coli* with the two phage vectors and followed the bacterial growth over time. We found that the growth of *E. coli* was independent of the phage used (Fig. 3a). Secondly, we titled the supernatant of the bacterial culture and found that the number of viruses in the M13K-

GFP culture was ~ 10 times less than the culture with the wildtype M13K07 (Table 2). Taken together, these data indicate that the newly generated M13K-GFP phage vector has maintained the ability to infect *E. coli* and form intact viral particles.

**Table 2:** The table reports the name, genotype, name of the foreign sequences added, the location of the foreign protein on the capsid, and concentration obtained after propagation of phage.

Name phage	Genotype	Displayed foreign protein	Location (on coat)	Titer in TU/mL
M13K07	Phage	None	None	3 × 10^12^
pLDP011	Phage vector	sfGFP	pIII	1 × 10^11^

We next sought to determine whether the sfGFP was functional. To do so, we conducted a fluorescent measure of the M13K-GFP phage vector by visualizing it under RGD light. We found that the phage solution containing the wildtype M13K07 did not show any fluorescence. In contrast, the phage solution containing the M13K-GFP showed a strong fluorescent signal indicating that the sfGFP gene, cloned in frame with *gene-3*, was transcribed into a functional protein (Fig. 3a–c). Taken together, these results indicate that the newly generated M13K-GFP is functional and can be easily visualized.

**Figure 4:**
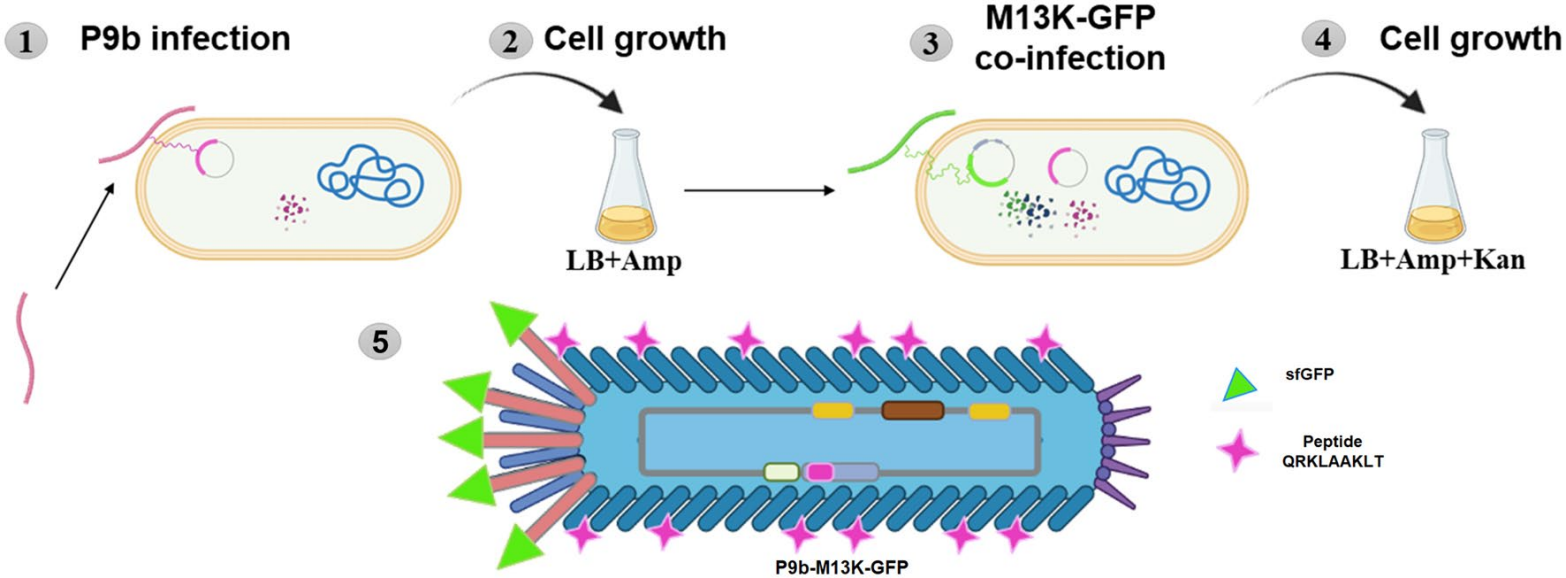
Schematic representation of the strategy used to generate the dual phage. Step 1: Infection of *E. coli* with P9b phagemid vector (Amp^+^). Step 2: Growth of transformed cell in LB media supplemented with Ampicillin. Step 3: Co-infection of transformed cell with M13K-GFP (Kan^+^) phage vector. Step 4: Growth in LB media supplemented with Ampicillin and Kanamycin. Step 5: Cartoon of phenotype of dual-display phage, P9b-M13K-GFP showing a hybrid structure of the pVIII proteins (some wild-type and some engineered with the foreign peptide QRKLAAKLT) and all the pIII proteins engineered with GFP.

The M13K-GFP phage vector can be clearly identified thanks to the expression of the GFP gene and therefore it could be utilized as a detection system when paired with another phage used to identify a specific target. To test this hypothesis, we utilized the P9b phagemid phage vector, which displays the foreign peptide QRKLAAKLT in-frame with pVIII. The expression of this fusion protein allows P9b to selectively recognize *Pseudomonas aeruginosa,* as we previously reported^19,29^. We used the M13K-GFP phage vector as a helper phage in the propagation of the P9b phagemid, thereby generating a dual display phage. To this end, we coinfected *E. coli* with both P9b and M13K-GFP; the former brings the modified engineered pVIII (with the peptide that targets *P. aeruginosa*) while the latter provides all the wildtype capsid proteins and the engineered pIII. The final product is the generation of a dual display phage (Fig. 4).

**Figure 5:**
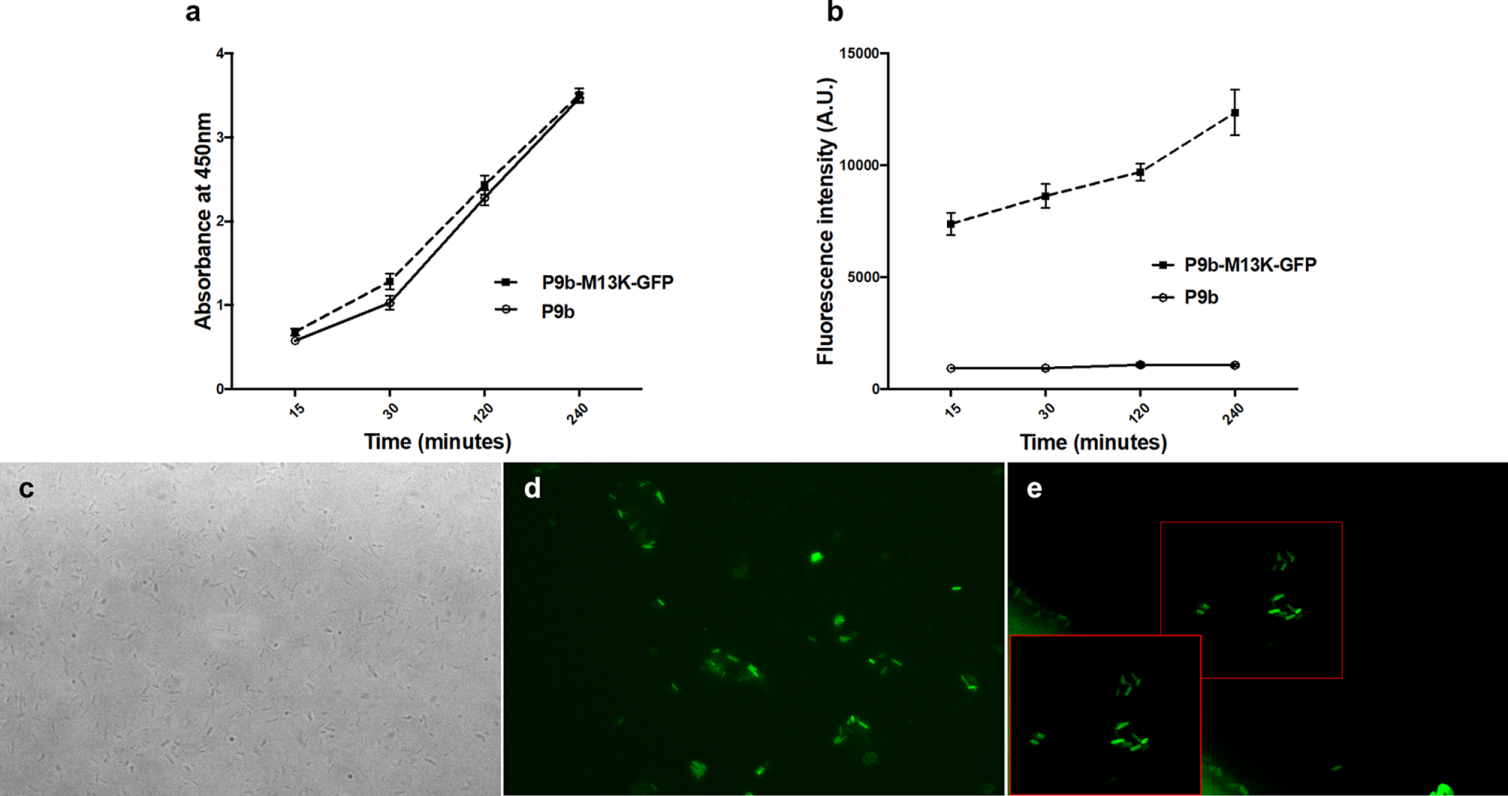
Validation of the dual phage. (**a**,**b**) Kinetic phage-capture ELISA results with the P9b or P9b;M13K-GFP against the bacterium target, *P. aeruginosa* detect by Anti-m13 antibody and by multilabel Vector 3 system. (**c**–**e**) Representative pictures of the P9b-M13K-GFP and *P. aeruginosa* one hour after incubation. Pictures were acquired in brightfield (**c**) and using FITC-filter (**d** and **e**). Data were analyzed by t-test. (**a**) p > 0.05; (**b**) p = 0.0037.

We next sought to determine whether the dual display phage maintained all the properties of the individual phages P9b and M13K-GFP. To this end, we added the dual display phage to *P. aeruginosa* and visualized the phages that identified the bacteria with a traditional ELISA and with GFP-mediated fluorescence. When comparing the detection by ELISA, we found that the signal emitted was similar between the dual phage display and the P9b alone, indicating that the dual phage display keeps the same binding efficiency to its target, *P. aeruginosa* (Fig. 5a). Next, to determine if the dual display phage also maintains the properties of M13K-GFP (i.e., emission at 488 nm), we first monitored the kinetic reaction with a multilabel fluorescent system and found that the dual display phage was easily detected by fluorescence (Fig. 5b). Subsequently, we also detected the phage interacted with the bacterial membrane using a simple fluorescent microscope (Fig. 5c–e). These data confirm that the dual display phages bind *P. aeruginosa* thanks to the peptide fuse to the pVIII, as we previously showed^29^, and it is easily detectable thanks to the GFP fuse to the pIII.

**Figure 6:**
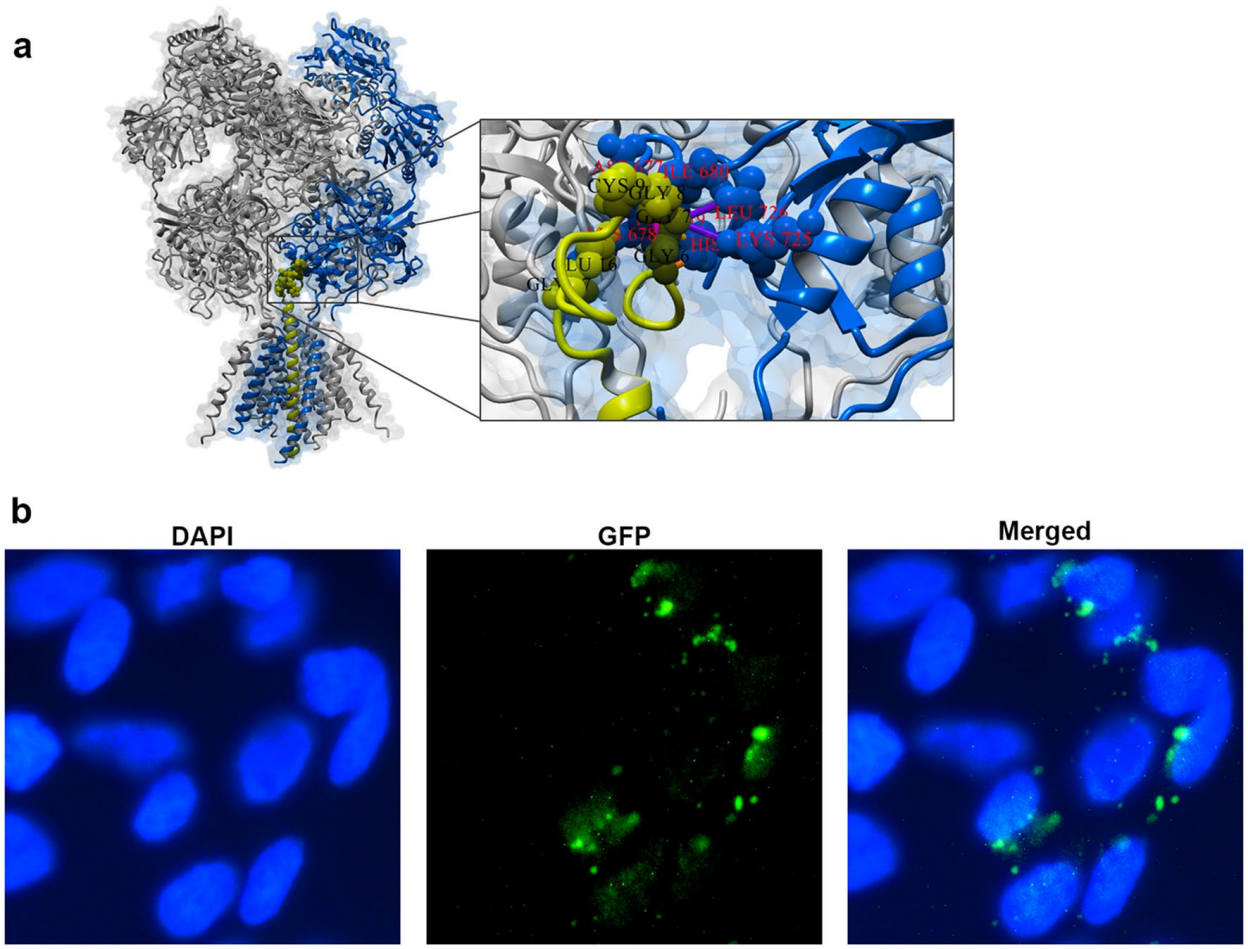
Use of the dual phage display with eukaryotic cells. (**a**) In silico 3D models of the interactions between NMDA receptors (PBD ID 6irh; gray and blue) and 12CIII1 phage (yellow). For each protein the amino acids involved in the interaction are displayed in the Ball&Stick style and labelled in black or red, for NMDA and pVIII-12CIII1, respectively. (**b**) Fluorescence microscopy images of SH-SY5Y incubated with the dual display phage.

M13K-GFP is a highly versatile phage that can be used to generate any kind of dual display phage. To this end, we next sought to determine whether our system could be used to detect eukaryotic cells. For this purpose, we combined the 12CIII1 phagemid vector, which displays the foreign peptide GGGCIEGPCLEG in-frame with pVIII^20^ and the M13K-GFP phage vector thereby generating a dual display phage. We have previously shown that the peptide of the 12CIII1 is a mimetic of amyloid-β (Aβ)^20^, a peptide that is thought to be crucial in the pathogenesis of Alzheimer’s disease^30^. Evidence from multiple laboratories has highlighted that Aβ physically interacts with the N-methyl-D-aspartate receptors^31–33^. Given that the foreign peptide exposed by the 12CIII1 phage is a mimetic of Aβ, we hypothesized that it could also interact with NMDA receptors. To test this hypothesis, we performed an in-silico 3D Modelling and Docking analysis. As expected, we found a strong interaction between the pVIII of the 12CIII1 phage and NMDA receptors (Fig. 6a). We then incubated the dual display phage with SH-SY5Y cells for 24 h before assessing the presence of the phage by fluorescence microscopy (Fig. 6b). We found that, in combination with a different phagemid, the M13K-GFP phage helper allowed the visualization of eukaryotic cells without the need for any type of signal amplification (Fig. 6b). Taken together, these data further highlight the versatility of the newly generated M13K-GFP phage vector.

## Discussion

Genetically modified bacteriophages are employed in several fields, such as in phage therapy as a source of new antimicrobials; in the development of biosensor devices for health and environmental purposes, and as genetic engineering tools themselves^34^. In this context, synthetic biology refers to the rational design, or de novo addition of functional genes according to the specific area of study. Genetic modification of the phage genome has been widely used to overcome limitations of natural bacteriophages^35,36^. For example, Kaźmierczak and colleagues recently cloned the red fluorescent protein gene with the hoc gene of the T4 phage. This resulted in the creation of a fluorescent phage, which can be observed in vivo in mice^36^.

Conversely, phage display systems have been instrumental in uncovering mechanisms of molecular interaction, particularly in the fields of antigen discovery, vaccine development, protein interaction, and cancer diagnosis and treatment^37–39^. In this case, exogenous peptides or entire antibodies of interest can be selected from a large library of phage particles by screening for affinity against desiderated targets^40^. The selected whole phage or partial phage particle can be used as a probe in phage-based biosensor platforms.

Traditionally, the phage-probe must be linked to a transducer, which converts the occurred interaction between phage and target into a detectable signal^41,42^. Several transducer systems have been used in combination with phage-probes. These include the detection of a colorimetric or luminescent signal by ELISA using an antibody against the phage or a phage labeled with fluorochromes^43,44^. Furthermore, viral DNA detection by PCR or physical signal by raman and sers-raman analysis have also been used. However, these transducer systems often make the process time-consuming and expensive^45,46^.

To overcome this limitation, in this study, we used the M13K07 helper phage as a backbone to design a fluorescent helper vector to use in combination with phagemid vectors thereby obtaining a dual display phage. We manipulated the M13K07 helper phage genome by Gibson assembly^28^. We performed a rational design and added the sfGFP gene in frame with *gene 3* of the phage capsid thereby obtaining a pIII-sfGFP fusion protein. Given the primary role of pIII in the physiology of the bacteriophage, erroneous modifications of its gene may interfere with the capsid formation or with the bacteriophage’s ability to infect *E. coli*. Notably, our modified phage helper generated vital phage particles packing the new helper phage vector. The lower yield of fluorescent helper phage particles quantified at the end of propagation can be attributed to the more time needed to amplify the whole genome of M13K-GFP and to assemble the capsid structure compared to the M13K07 wild-type. Furthermore, the ability of M13K-GFP to emit fluorescence when excited at 535 nm confirmed that the sfGFP fused to the pIII maintained the correct conformation to generate fluorescence^47^.

Using M13K-GFP as a helper phage in an 8 + 8 phage display system, we obtained a dual display phage. In the classical scheme, the M13K07 helper phage, which carries all the genes needed for infection, replication, assembly, and budding, is used for packaging phagemid in *E. coli*. Phagemid vectors contain three key elements (i) an antibiotic marker (ii) the gene encoding the pVIII-fusion protein with a specific peptide, and (iii) the regions of phage origin of replication. Upon co-infection, wild-type pVIII from the helper phage competes with the phagemid encoding the pVIII-peptide fusion protein for incorporation into the phage progeny obtaining phage particle with hybrid capsid in the pVIII with fused peptide and wild-type^14^.

M13K-GFP contains all the genes needed to generate a complete phage particle and the sfGFP gene fused to *gene 3*. When we combined it with phagemids P9b we obtained a dual display phage with a hybrid capsid having wildtype pVIII and pVIII fused to a peptide specific for *P. aeruginosa*. When we combined M13K-GFP with 12CIII1, we obtained a dual display phage with a peptide fused to the pVIII specific for the eukaryotic SH-SY5Y cells. In both cases, the dual display phage expressed the sfGFP fused to the pIII. Notably, our newly generated dual display phage can be visualized with both a multilabel plate reader or a fluorescence microscope, without the addition of any other antibody or molecule to amplify the signal. While this system may render the propagation of the phage more laborious (i.e., there will always be a need to start from a phagemid and the GFP help phage), it provides significant versatility. One can readily combine M13K-GFP with a phagemid vector of their choice, allowing for the display of a specific peptide on the pVIII. Overall, by coupling synthetic biology methods with phage display systems, we obtained a dual display phage particle that can be used for a variety of in vitro and in vivo applications. In addition, we anticipate that multifunctional phage particles can increase the use of engineered phages in the development of greatly simplify the detection steps.

## Materials and methods

### Bacterial strains

*Escherichia coli* MG1655 was used for cloning and plasmid production. *E. coli* TG1 (F+, lacZ^−^, Amp^−^, Kan^−^) was used to check infectivity and to amplify wild-type and engineered phage. Both *E. coli* strains were propagated in Luria Bertani broth (LB) and Luria Bertani Agar (LA) substrates. Stock organisms were maintained in LB containing 20% (v/v) glycerol at − 80 °C.

### Bacteriophage and plasmid

Filamentous M13K07 helper phage was used as the backbone for the cloning reaction. It derived from an M13 wild-type phage modified to express a kanamycin resistance gene (Kan^+^) and a p15A origin of replication. It is used in the phage display technique as a helper in co-infection with a phagemid vector system.

P9b and 12CIII1 phagemid vectors were used to generate the dual-display phage. 12CIII1 derived from the M13-pVIII-9/12aa peptide library as previously described^19,29^. The clones display the foreign peptide QRKLAAKLT and GGGCIEGPCLEG respectively, in frame to pVIII. Their genotype is based on the Ampicillin resistance gene (Amp^+^), phage origin of replication (ORI), phage-packaging region of DNA from the phage M13-genome, and gene 8 with *in frame* the nucleotide sequences that transcribe for foreign peptide. Moreover, the promoter Isopropyl ß-d-1-thiogalactopyranoside (IPTG) and the LacZ gene were also present upstream and downstream of gene 8, respectively. This phagemid vector system can be converted to filamentous phage particles by co-infection with the helper phage, such as M13K07, assuming phenotype as M13 phage hybrid in pVIII proteins capsid. All filamentous phages were maintained in Tris-buffered saline (TBS, 7.88 g/L of Tris hydrochloride, and 8.77 g/L of NaCl in deionized water). The plasmid contained the *sfGFP* gene, Kan^+^ and the pet-R promoter was maintained in the transfected *E. coli* MG1655.

### Preparation of genetic materials

A batch of heat-shock-competent *E. coli* MG1655 was transformed with the sfGFP plasmid. *E. coli* TG1 broth culture at optical density (OD_600nm_ = 0.7) was infected with M13K07 and then incubated at 37 °C in static condition for 15 min, followed by shaking at 250 rpm for 20 min. To obtain the DNA template for Phusion PCR, we selected one colony and extracted its DNA using the QIAprep Spin Miniprep Kit (Qiagen), following the manufacturer’s instructions.

### Amplification of overlapping DNA fragments using Phusion PCR

The primers were designed using the Snapgene tool (Table 1). The PCR mixture contained 10 µl of 5X HF buffer, 2.5 μl of each primer, 1 μl of dNTPs mix (10 mM each) and 0.5 μl of Phusion^®^ High-Fidelity DNA polymerase (New England BioLabs). PCR reactions contained approximately 10 ng of template plasmid and were carried out under the following conditions: the initial denaturation of 30 s at 98 °C, followed by 40 cycles of 10 s at 98 °C, 30 s at 60 °C, 30 s per kb at 72 °C, followed by a final elongation of 10 min at 72 °C. In each tube of reaction 5 μL of CutSmart buffer, and 0.5 μL of DpnI enzyme were added and incubated at 37 °C for 1 h. Subsequently, 5 μL of amplified fragments was added to 2 μL of run-leader and analyzed by 1% agarose gel electrophoresis (1% wt/vol agarose in 1X TAE buffer). The amplified fragments were purified by Nucleospin Gel and PCR clean-up (Qiagen), according to the manufacturer’s instructions. Each fragment was amplified and processed in duplicate, and then collected and quantified by Nanodrop.

### In vitro recombination by Gibson assembly

The purified fragments were assembled according to the one-step isothermal DNA assembly method described developed by Gibson and colleagues^28^. Fragments were pooled in 5 µL (to have 300 ng total fragments, in equimolar concentration) and add to 15 µL of Gibson mix. Tubes were incubated in the thermocycler at 50 °C for 1 h. Gibson-product was dialyzed for 30′ on a filter (0,025 µm membrane MCE), previously soaked in deionized water. Three µL were used to transform 20 µL of competent *E. coli* MG1655 by electroporation. The mixture was then transferred to a 2 mL tube with 1 ml of S.O.C. medium and incubated at 37 °C for 1 h, shaking at 200 rpm, before to plate on LA with Kan.

### Colony PCR and sequencing

13 positive colonies were randomly chosen and cultured in LB media with Kan antibiotic at 37 °C, and diluted in 50 µL dd-H_2_O to perform colony PCR. 8 µL of the diluted colony was added to 10 µL Dream taq mix (2X) with 1 µL of each primer, set FW: 5′-GCTACCCTCGTTCCGATGCTG-3′ and RV: 5′-CCGCCAGCATTGACAGGAG-3′ to check correct assembly of phage helper fragments (size 1663 bp); and set FW: 5′-GGACGGCAACATCCTGGGC-3′ and RV: 5′-CCGCCAGCATTGACAGGAG-3′ to check for the presence of sfGFP (size 959 bp). The thermocycling conditions included an initial denaturation at 95 °C for 3-min, followed by 30 cycles comprising of 95 °C for 30-s, 60 °C for 30-s, and 72 °C for 70-s, and one final elongation step at 72 °C for 5-min. PCR products were purified by NucleoSpin PCR Clean-up purification Kit (Macherey–Nagel) and sent for sequencing using the forward primer.

### Formation of viable M13K-GFP phage

The transformed colonies were picked and inoculated in LB containing 50 µg/mL Kanamycin and incubated overnight at 37 °C while shaking. Phages (if present) were collected as previously described^17^. Briefly, the culture was centrifuged at 8000×*g* for 20 min at 25 °C. The supernatant was recovered and mixed with 25% (v/v) of PEG/NaCl solution and cooled in ice for 4 h. Then it was centrifuged at 15,300×*g* for 1 h at 4 °C. The pellet was recovered and resuspended in 10% (v/v) of TBS mixed with 25% (v/v) of PEG/NaCl, cooled on ice for 4 h, and the solution was centrifuged again as above. The pellet (containing phage particles) was suspended in 10% (v/v) of TBS, filtered through 0.22 μm pore size membrane (Millipore) and stored at 4 °C. 10 μL of the solution was used to infect the exponentially growing *E. coli* TG1 cells by incubation it at 37 °C for 15 min in static condition and 20 min in agitation. After incubation, samples were dispensed into LA plates containing 50 μg/mL kanamycin and incubated at 37 °C overnight. Finally, the solution was analyzed in the fluorescence emission by Fluorescent Imaging System under RGD light (UP), confocal-fluorescent microscopy using a FITC filter and by multiscan Vector 3 system.

### M13K07 and M13K-GFP phage vector propagation

One colony of *E. coli* strain TG1 infected with M13K-GFP or M13K07 phage vectors was inoculated into 20 mL of LB containing 50 μg/mL kanamycin and incubated at 250 rpm on a rotary shaker at 37 °C until it reached an OD_600_ of 0.2. Then the solution was transferred to a flask containing 250 mL of LB containing 50 μg/mL kanamycin and incubated at 37 °C overnight.

### Phagemid vector and dual-phage generation

*Escherichia coli* TG1 was infected with P9b or 12CIII1 phagemid vector and incubated at 37 °C, in static condition for 15 min and at 250 rpm on a rotary shaker for 20 min. After incubation, infected *E. coli* was plated onto LA plates containing 50 μg/mL ampicillin and incubated overnight at 37 °C. The next day, one colony of *E. coli* containing phagemid vector was inoculated into 20 mL of LB containing 50 μg/mL ampicillin and incubated at 250 rpm on a rotary shaker at 37 °C until the OD_600_ reached 0.2. Subsequently, 40 μg/mL IPTG, M13K07 or M13K-GFP helper phages were added to the colony (10^9^ transduction units per milliliter, TU/mL), which was incubated at 37 °C, in static condition for 15 min and at 250 rpm on a rotary shaker for 20 min. The colony was then transferred to a flask containing 250 mL of LB with 50 μg/mL Kanamycin and Ampicillin and incubated at 37 °C overnight. Phages were also quantified by tittering (TU/mL) as described in^48^.

### Phage ELISA

100 μl of suspensions of *P. aeruginosa* in exponential growth (OD 540 = 0.8) were coated 96-well ELISA plates overnight at 4 °C in carbonate buffer (35 mM NaHCO3, 15 mM Na_2_CO_3_, pH 9.6); washed three times with washing buffer (0.05% Tween 20 in PBS); blocked with Blocking buffer (6% non-fat dry milk, 0.05% Tween 20 in PBS) for 2 h at 37 °C, washed again (five times); incubated with 100 μl of phage, (P9 and P9b-M13K-GFP 10^11^ TU/ml) at different times 15 min, 30 min, 60 min and 120 min at 37 °C with shaking, washed again (five times); and re-incubated with 100 μl of anti-pVIII-M13-HRP conjugate antibody at a dilution of 1:2500 in dilution buffer (1% non-fat dry milk, 0.05% Tween 20 in PBS) for 1 h at 37 °C, washed again (five times) after which the reaction was developed with 100 μl of TMB for 45 min at room temperature. Lastly, the reaction was stopped with 100 μl of 1 M H_2_SO_4_. Optical absorbance was recorded at 450 nm (Multiscan FC).

### Sample preparation for fluorescence imaging

100 μl of suspensions of *P. aeruginosa* (2 × 10^7^ CFU/mL) were incubated with 100 μl of phage (P9b-pLDP011, 1 × 10^9^ TU/ml) for 1 h at 37 °C. The mixture was then centrifuged at 8000×*g* for 20 min at 25 °C, the supernatant was removed to eliminate the unbound phages. The pellet was suspended in an iso-volume of PBS solution. The same process was performed without the addition of phages, as negative control. The samples were analyzed by a fluorescence microscope (LeicaDMRE) with 63× and 100× magnification.

### In silico 3D modelling and docking

Engineered pVIII protein structure modeling was performed using the MODELLER 9.20. The models were built as previously reported^17^. Briefly, the structure of pVIII protein was obtained from RCSB Protein Data Bank (PDB ID: 2mjz). Amino acid sequence of engineered pVIII protein of 12CIII1 clone were written on the following FASTA format to obtain the pVIII-engineered model 1-AEGEF**GGGCIEGPCLEG**DPAKAAFNSLQASATEYIGYAWAMVVVIVGATIGIKLFKKFTSKAS-64 (the sequence of the foreign peptide is in bold). The best 3D model was selected according to the lowest DOPE value, which indicates the construction energy. The pVIII-12CIII1 protein and NMDA 3D structure collected from RCSB Protein Data Bank (PDB ID 6irh) were used as template in a docking analysis using ZDOCK server. The best 3D model was selected, according to the Z-scope and opened using Yasara software to acquire the picture and the interaction between amino-acids residues.

### Cell culture

Human neuro-blastoma SH-SY5Y cells were obtained from American Type Culture Collection (ATCC CLR-2266) and were grown to a monolayer in a culture medium containing Dulbecco’s minimal essential medium (DMEM) and Ham’s F12, modified with 2 mM l-glutamine and 1.0 mM sodium pyruvate and supplemented with fetal bovine serum (FBS) to 10% and streptomycin 50 mg/mL. SH-SY5Y cells were maintained at 37 °C and 5% CO_2_.

For the immunofluorescent experiments, cells were seeded into round coverslips pretreated with poly-l-lysine in a 12-well plate (~ 100.000 cells/well) and incubated at 37 °C with 5% CO_2_ until cells reached a ~ 70% confluency. Cells were then incubated with ~ 1.0 × 10^11^ dual-phage for 24 h, after which they were fixed with 4% paraformaldehyde in PBS for 10 min at room temperature. After 2 washes with 1X PBS, cells were permeabilized with 0.1% Triton X-100 in PBS for 15 min at room temperature and blocked with 2% BSA for 30 min at room temperature. After washing, round coverslips were cover-slipped with VECTAshield mounting medium with DAPI (Vector Laboratories) and visualized by fluorescence microscopy using FITC and DAPI filters.

### Statistical analysis

Statistical analysis was performed using GraphPad InStat version 3.1. Non-parametric ANOVA and multiple comparison tests were used. The levels of statistical significance were as follows: p. < 0.0001 (****); 0.0002 (***); 0.0021 (**); 0.0332 (*).

## Data Availability

The datasets used and/or analysed during the current study available from the corresponding author on reasonable request.
